# Probabilistic Segmentation of Mass Spectrometry (MS) Images Helps Select Important Ions and Characterize Confidence in the Resulting Segments* [Fn FN2]

**DOI:** 10.1074/mcp.O115.053918

**Published:** 2016-01-21

**Authors:** Kyle D. Bemis, April Harry, Livia S. Eberlin, Christina R. Ferreira, Stephanie M. van de Ven, Parag Mallick, Mark Stolowitz, Olga Vitek

**Affiliations:** From the ‡Department of Statistics and; §Department of Chemistry, Purdue University, West Lafayette, IN 47907;; ¶Canary Center at Canary Foundation, Stanford University School of Medicine, Palo Alto, CA 94304; College of Science and; **College of Computer and Information Science, Northeastern University, Boston, MA 02115

## Abstract

Mass spectrometry imaging is a powerful tool for investigating the spatial distribution of chemical compounds in a biological sample such as tissue. Two common goals of these experiments are unsupervised segmentation of images into newly discovered homogeneous segments and supervised classification of images into predefined classes. In both cases, the important secondary goals are to characterize the uncertainty associated with the segmentation and with the classification and to characterize the spectral features that define each segment or class. Recent analysis methods have focused on the spatial structure of the data to improve results. However, they either do not address these secondary goals or do this with separate post hoc procedures.

We introduce spatial shrunken centroids, a statistical model-based framework for both supervised classification and unsupervised segmentation. It takes as input sets of previously detected, aligned, quantified, and normalized spectral features and expresses both spatial and multivariate nature of the data using probabilistic modeling. It selects informative subsets of spectral features that define each unsupervised segment or supervised class and quantifies and visualizes the uncertainty in spatial segmentations and in tissue classification. In the unsupervised setting, it also guides the choice of an appropriate number of segments. We demonstrate the usefulness of this framework in a supervised human renal cell carcinoma experimental dataset and several unsupervised experimental datasets, including a pig fetus cross-section, three rodent brains, and a controlled image with known ground truth. This framework is available for use within the open-source R package Cardinal as part of a full pipeline for the processing, visualization, and statistical analysis of mass spectrometry imaging experiments.

Mass spectrometry imaging visualizes the spatial distribution of molecular ions in a sample by repeatedly collecting mass spectra across its surface. Equipped with a computer-controlled sample stage, a mass spectrometer rasters across the sample and collects individual mass spectra from discrete or continuous locations. The leading technologies for performing MS imaging include matrix-assisted laser desorption/ionization (MALDI) and desorption electrospray ionization (DESI)^1^. MALDI imaging requires the application of a matrix solution and is typically used to detect large molecules such as peptides and proteins. DESI imaging does not require a matrix and is typically used to detect small molecules such as lipids, metabolites, and drug molecules (19). The intensities at a particular *m/z* value and spatial location can then be plotted as pixels in a false-color image, called an ion image, that displays the spatial distribution of the analyte associated with that *m/z* value. MS imaging has been shown to be promising in a wide range of biological applications such as molecular histology of tissue, whole body sections, bacterial films, etc. (17) and biomedical applications such as cancer diagnosis (7, 8).

Computational analysis of MS imaging experiments typically consists of preprocessing, followed by a statistical analysis (1). The preprocessing ensures that mass spectral intensities are comparable across all mass spectra in the experiment. This is typically done via normalization and (if necessary) baseline reduction. Furthermore, the preprocessing extracts spectral features that correspond to the underlying analytes. This is typically done via peak picking, or *m/z* binning or resampling. Much progress has already been made in the preprocessing of MS imaging data. Many mature tools for preprocessing mass spectra already exist (21). However, a major bottleneck is the downstream statistical analysis of the processed data. This manuscript focuses on the downstream statistical analysis steps, which take place after the detection, quantification, alignment, normalization, and (optionally) identification of the initial set of high-quality spectral features.

## 

### 

#### Related Work

Two common primary goals of statistical analysis of MS imaging experiments post signal processing are classification (for supervised experiments), *i.e.* assigning pixels to predefined classes, and segmentation (for unsupervised experiments), *i.e.* assigning pixels to newly discovered segments. A number of methods for these purposes already exist.

Traditional multivariate methods are frequently used for both classification and segmentation. For classification, methods including linear discriminant analysis, projection to latent structures discriminant analysis (PLS-DA), and orthogonal projection to latent structures discriminant analysis (O-PLS-DA) have proven effective (8, 7, 10, 11, 16, 20). For segmentation, clustering methods such as hierarchical clustering or k-means (sometimes preceded by principal components analysis to reduce the dimensionality of the spectra) are frequently used (6, 12, 13). The traditional multivariate methods have two drawbacks. First, they are agnostic of the spatial structure of the data. They treat each pixel independently and ignore similarities of spectra acquired from spatially proximate locations, thereby compromising the accuracy of the results. Second, they do not reduce the input features to more informative subsets, thereby compromising the interpretation.

Several recent methods were specifically designed to account for the spatial structure of MS images. One family of methods relies on the spatial structure to detect quality peaks from raw spectra (2, 18). Although highly valuable, these methods stop at processing the signals and do not address the goals of image segmentation or image classification. Another family of methods, including spatially aware clustering and spatially aware structurally adaptive clustering by Alexandrov and Kobarg (4), account for the spatial structure of the data and demonstratively improve the quality of image segmentation (1). However, similarly to the multivariate analysis methods, they do not select subsets of spectral features that define the segments and rely on post hoc techniques to interpret the features associated with the segments, *e.g.* using the Pearson correlation between a segment and the single ion images (1, 13).

On the other hand, statistical regularization has become a method of choice for extracting subsets of informative features from highly multivariate data. One such method is nearest shrunken centroids introduced by Tibshirani *et al.* (14, 15), which was originally developed for classification of gene expression microarrays. A related method has been applied to classify tissues in MS imaging experiments using regularized logistic regression (9). However, similarly to the multivariate analysis methods, they do not account for the spatial structure of the data.

#### Contribution of the Manuscript

This manuscript contributes a general statistical modeling and inference methodology for unsupervised segmentation and supervised classification of MS images. It takes as input a set of previously detected, quantified, aligned, and normalized features, produced by any signal processing method of choice. It combines the advantages of both spatially aware clustering by Alexandrov and Kobarg and statistical regularization by Tibshirani *et al.* We show that, for unsupervised segmentation, the spatial probabilistic modeling provides better quality segmentation. It characterizes the probability of segment membership for each pixel and allows us to quantify and visualize the uncertainty of segmentation for each pixel. Moreover, statistical regularization aids interpretation by automatically selecting subsets of the spectral features, such that each subset defines each segment. Statistical regularization also enables data-driven selection of the number of segments. Similarly, for supervised image classification probabilistic modeling characterizes the probability of predefined tissue class membership for each pixel and aids interpretation by automatically selecting subsets of spectral features that define each class.

We evaluate the performance of supervised classification on a human renal cell carcinoma experiment, demonstrating its utility for diagnosing pixels as cancer or normal tissue. We evaluate the performance of unsupervised segmentation on several biological samples, including a pig fetus cross-section and three rodent brain datasets, demonstrating its utility for studying morphology. For unsupervised segmentation, we also evaluate on a nonbiological sample of known composition in order to test its accuracy under controlled conditions. This framework has been previously implemented in the open-source R package Cardinal (5), as part of a full pipeline for the processing, visualization, and statistical analysis of mass spectrometry imaging experiments. This manuscript contributes the theoretical framework behind this implementation.

## EXPERIMENTAL PROCEDURES

### 

#### 

##### Unsupervised Segmentation: Pig Fetus Cross-Section

**Fig. 1.**
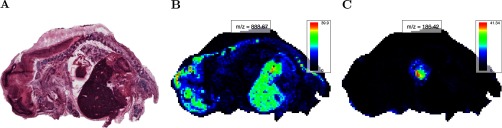
**Pig fetus cross-section: morphology and single ion images.** (*A*) Optical image of H&E-stained pig fetus cross-section showing its morphology, including the brain (*left*), heart (*center*), and liver (dark region below heart). (*B* and *C*) Characteristic ion images for the pig fetus dataset at (*B*) 888.67 *m/z*, showing the brain and liver, and (*C*) 186.42 *m/z*, showing the heart.

The primary goal of this experiment was to discover morphological features of the pig fetus, such as major organs, through unsupervised analysis of the mass spectra. A secondary goal was to find spectral features associated with the morphological features. Fig. 1*A* is an optical image of the Hematoxylin and eosin (H&E)-stained tissue section showing the general morphology of the pig fetus, including major organs such as the brain, heart, and liver.

Mass spectra were collected using a Thermo Finnigan LTQ linear ion trap mass spectrometer with a DESI ion source over the 150–1,000 *m/z* range. The images were cropped to remove non-informative spectra originating from the glass slide. The cropped dataset consisted of 4,959 mass spectra with 10,200 spectral features. The mass spectra were normalized to a common total ion current, and peak picking was performed to reduce the dataset to 143 peaks. All data processing and analysis were performed using Cardinal (5).

Fig. 1*B* shows a single ion image featuring the brain and liver, and Fig. 1*C* shows a single ion image featuring the heart. Below, we will use this dataset to demonstrate unsupervised statistical analysis using all the mass spectral peaks to recover the major morphological features.

##### Unsupervised Segmentation: Cardinal painting with Known Segmentation

The goal of this experiment was to use a controlled sample to evaluate the quality of data acquisition and statistical analysis. A painting of a cardinal on paper was affixed to a glass slide, and MS imaging was applied. An optical image of the cardinal painting during data acquisition is shown in Supplemental Fig. 1*A*.

The mass spectra were acquired using a Thermo Finnigan LTQ linear ion trap mass spectrometer with a DESI ion source over the 100–1,000 *m/z* range. The dataset consisted of 12,600 mass spectra with 10,800 spectral features. Mass spectra were normalized to a common total ion current, and peak picking was performed to reduce the dataset to 51 peaks. All data processing and analysis were performed using Cardinal.

Supplemental Fig. 1*B* shows a single ion image featuring the “DESI-MS” text part of the painting, and Supplemental Fig. 1*C* shows a single ion image featuring the red pigment used in the cardinal body. The painting itself shown in Supplemental Fig. 1*A* can be considered the ground truth image. We use this dataset to evaluate the ability of the unsupervised statistical analysis of the mass spectral peaks to recover the ground truth.

##### Unsupervised Segmentation: Rodent Brain Images of Varying Quality

The goal for these datasets is to compare the results of several similar experiments of varying data quality. All three experiments involved a rodent brain.

**Fig. 2.**
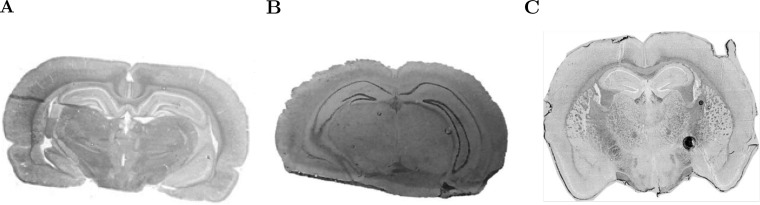
**Rodent brain morphologies.** (*A*) Optical image of rat brain (R1). (*B*) Optical image of mouse brain (R2). (*C*) Optical image of mouse brain (R3).

The first dataset (R1) is a high-quality image of a rat brain (3, 4), which is shown in Fig. 2*A*. Mass spectra were acquired on a Bruker Autoflex III MALDI-TOF mass spectrometer over the 2,500 to 25,000 *m/z* range. The images were cropped to remove noninformative spectra, and only the 2,500 to 10,000 *m/z* range was used. The reduced dataset consisted of 20,185 mass spectra with 3,045 spectral features. The mass spectra were normalized to a common total ion current, and baseline correction was performed using ClinProTools. Cardinal was thereafter used to perform peak picking to reduce the dataset to 80 peaks. Except for baseline correction and normalization, all data processing and analysis were done in Cardinal.

The second dataset (R2) is a mouse brain shown in Fig. 2*B*. This experiment produced high-quality spectra but with a moderate amount of experimental noise. Mass spectra were acquired on a Thermo Finnigan LTQ linear ion trap mass spectrometer with a DESI ion source over the 200–1,000 *m/z* range. The images were cropped to remove noninformative spectra. The cropped dataset consisted of 8,950 mass spectra with 9,600 spectral features. The mass spectra were normalized to a common total ion current, and peak picking was performed to further reduce the dataset to 123 peaks. All data processing and analysis were done in Cardinal.

The third dataset (R3) is a mouse brain shown in Fig. 2*C*. This dataset features a high degree of experimental noise. Mass spectra were acquired using an AB Sciex MALDI TOF/TOF 5800 System over the 4,000 to 20,000 *m/z* range. The images were cropped to remove noninformative spectra. The cropped dataset consisted of 4,923 mass spectra with 22,667 spectral features. The mass spectra were normalized to a common total ion current, smoothed, and baseline corrected. Peak picking was then performed to reduce the dataset to 57 peaks. All data processing and analysis were done in Cardinal.

We will use these datasets to characterize the ability of the results of statistical analysis to reflect differences in data quality.

##### Supervised Segmentation: Human Renal Cell Carcinoma

The goal of this experiment was to classify renal tissue specimens as cancer or normal. In accordance with approved Institutional Review Board protocols at Indiana University School of Medicine, matched pairs of tissue were collected from human subjects with renal cell carcinoma (RCC), with each pair consisting of cancerous tissue and adjacent normal tissue (8). Supplemental Fig. 2 shows optical images of the eight tissue pairs we analyzed. Each tissue was manually annotated as normal or cancerous by a pathologist. However, the annotations are based on the dominant tissue type for each whole tissue, so some tissues may contain regions from the nondominant class.

The mass spectra were collected using a Thermo Finnigan LTQ linear ion trap mass spectrometer with a DESI ion source over the 150–1,000 *m/z* range. The images were cropped to remove noninformative spectra originating from the glass slide. The cropped dataset consisted of 6,077 mass spectra with 10,200 spectral features. Individual tissue samples consisted of between 972 to 3,564 mass spectra per matched pair. The mass spectra were normalized to a common total ion current and resampled to unit resolution, resulting in 850 spectral features. All data processing and analysis were performed using Cardinal. Supplemental Fig. 3 shows single ion images for 215.3 *m/z*, which is an ion known to be more abundant in normal tissue, and Supplemental Fig. 4 shows single ion images for 885.7 *m/z*, which is known to be more abundant in cancerous tissue (8). Some tissues appear to exhibit heterogeneity, such as the abundance of 215.3 *m/z* along the edge of the cancerous tissue in sample UH0505 12 (Supplemental Fig. 3*B*). We will use this dataset to demonstrate the ability of the proposed framework to perform classification, while selecting spectral features important in distinguishing the disease condition.

## RESULTS

### 

#### 

##### Overview and notation

Let *m* = 1, …, *M* denote the index of the biological sample, *i.e.* a slide with one (or several) tissues. On slide *m*, the experiment collects *N_m_* spectra at *N_m_* total pixel locations. Therefore, over all the samples, the experiment contains *N* = Σ_*m*=1_^*M*^ *N_m_* spectra and pixels.

Let (*i, j*) denote the location of a pixel on a sample *m*. We do not assume that the samples are rectangular in shape, so the indices (*i, j*) are arbitrary. However, we do assume (*i* + δ*_i_*, *j* + δ*_j_*) describes the location of a pixel (δ*_i_*, δ*_j_*) away on the same sample. We assume that the spectra acquired at these locations have been processed, so that every spectrum has the same *P* features, defined as a picked peak or a binned *m/z* range. We also assume that the pixel intensities are normalized so that spectra are comparable across pixels and across samples. Then, denote the spectrum acquired at a pixel location (*i, j*) on sample *m* as x*_ijm_* = {x*_ijmp,_ p* = 1, …, *P*}. In other words, spectrum x*_ijm_* is a vector of scalar intensities *x_ijmp_* for *P* spectral features.

Suppose also that the spectra and the pixels belong to one of *K* classes (for supervised classification) or segments (for unsupervised segmentation). For supervised classification, the class membership is known, for example, from annotation by a pathologist, and the statistical goal of the experiment is to classify each pixel to one of these classes in a supervised manner based on its spectrum. Alternatively, for unsupervised segmentation, the class membership is unknown, and the statistical goal of the experiment is to discover these classes from the spectra in an unsupervised manner. Let *N_k_* denote the number of spectra, and the number of pixel locations, assigned to class *k* = *1*, …, *K* by an unsupervised or a supervised procedure.

Additionally, we denote the mean spectrum for a known class or discovered segment *k* as x̄*_k_* and the overall mean spectrum as x̄. That is, x̄*_k_* is a vector of *P* scalar intensities *x̄_kp_*, which are the mean intensities for spectral feature *p*, over spectra from all pixel locations assigned to class *k*, and x̄ is a vector of *P* scalar intensities *x̄_p_*, which are the overall mean intensities for spectral feature *p*, over all spectra.

In the following section, we discuss the proposed spatial shrunken centroids framework for both supervised classification and unsupervised segmentation. For supervised classification, the method relies on the known classes. For unsupervised segmentation, where the segments are unknown, the segments are initialized randomly or by another segmentation method such as spatially-aware clustering (4) and are updated over multiple iterations until one of several convergence criteria is met. We detail the important steps below. The full algorithms are available in Supplementary Section 2.1.1 and Supplementary Section 2.1.2.

##### Proposed Statistical Framework for Supervised Classification and Unsupervised Segmentation of Mass Spectrometry Images

##### Characterization of Classes and Segments by Their Shrunken Centroids

In mass spectrometry, imaging tissue region, condition, or class is typically summarized by a mean spectrum, also called its centroid, x̄*_k_*. Here, we propose that each class (or segment) is better represented using shrunken centroids, from the method of nearest shrunken centroids by Tibshirani *et al.* (14, 15). This will allow us to compare the class (or segment) centroids to the overall centroid and to select the informative spectral features (defined as being very dissimilar to the overall centroid). We detail this below.

We follow Tibshirani *et al.* and calculate the class (or segment) centroids, x̄*_k_* and use statistical regularization to shrink the centroids toward the overall centroid x̄. We then calculate the *t*-statistic for spectral feature *p* for class (or segment) *k* as

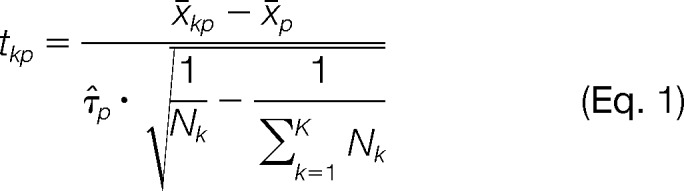
 Here τ̂*_p_* is the pooled estimate of the within-class standard deviation for feature *p*. The number $\sqrt{\frac{1}{N_{k}}-\frac{1}{\sum_{k=1}^{K}N_{k}}}$ makes the denominator equal to the estimated standard error of the numerator. Second, we apply the soft thresholding operator ()_+_ to shrink the *t*-statistics toward 0.

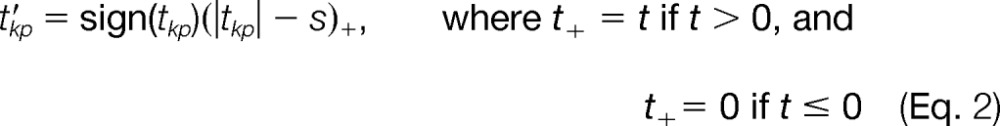
 and *s* is the shrinkage parameter. Larger values of *s* lead to a larger number of *t*-statistics *t*′_kp_ to be set to 0. Finally, we define the intensities of the shrunken centroids for each feature *p* for each class (or segment) *k* as

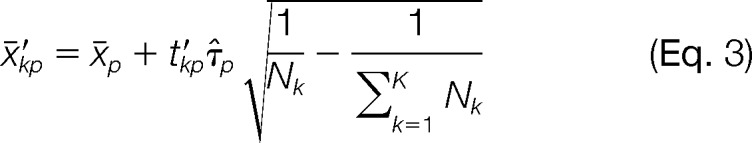
 so that *x̄′_k_* = {x′*_kp_* = *1*,…, *P*} is the shrunken centroid for class (or segment) *k*.

The shrunken centroids x̄′*_k_* here can be viewed as adjusted mean spectra of the *K* classes (or segments), where the intensities have been adjusted toward the overall mean spectrum. Therefore, the characteristic mean spectrum for a class (or segment) should differ from the overall mean spectrum only for those spectral features that are truly characteristic of the class (or segment). Spectral features that are not meaningfully different from the overall mean spectrum will have intensities set to the overall mean intensity for that feature.

##### Selection of Informative Features

The shrunken t-statistics *t′_kp_* calculated in Equation 2 are well suited for selecting informative features. The spectral features with *t′_kp_* > 0 are systematically enriched for class (or segment) *k*. Likewise, spectral features with *t′_kp_* < *0* are systematically absent from class (or segment) *k*, as compared with the overall mean spectrum. Spectral features with *t′_kp_* = 0 are noninformative, as only the features with *t′_kp_* ≠ 0 matter when assigning a pixel's spectrum to class (or segment) *k*.

##### Spatially Aware (SA) and Spatially Aware Structurally Adaptive (SASA) Distances

To classify the individual pixel or to assign a pixel to a segment, we need to define a distance between the spectra from individual pixels and the shrunken centroids. We propose to use the spatially aware distance defined by Alexandrov and Kobarg (4). We detail this method below and show how we adapt it in the section Defining the SA and SASA Distances to the Shrunken Centroid of a Class or Segment.

Alexandrov and Kobarg proposed a spatially aware distance between two spectra x*_ijm_* and x*_i′j′m_*, which depends on the spectra from pixels within a neighborhood of (*i, j*) and ([i′, j′]). The authors showed that this approach is beneficial, as it produces better quality segmentations, as compared with naïve methods that do not account for the spatial relationships between pixels (3). Therefore, for a neighborhood radius of *r*, the distance between two spectra is defined as

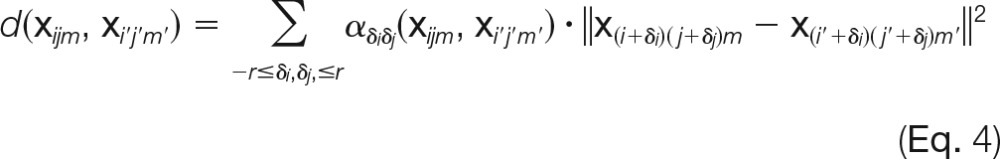


Here, the α_δ*_i_*δ*_j_*_(*X_ijm_*, *X_i′j′m′_)* are spatial weights of the neighbors. The exact definition of these weights results in either a spatially aware (SA) distance or a spatially aware structurally adaptive (SASA) distance. In the SA distance, the weights are defined as

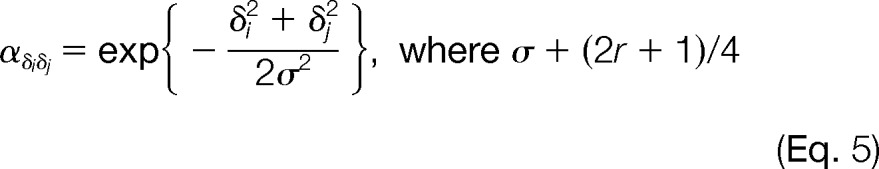
 which are Gaussian weights independent of the spectra and only depend on the neighborhood. Using Gaussian weights, which decrease with the distance δ_*i*_^2^ + δ_*i*_^2^ from the neighborhood center, is a natural choice because it assumes that pixels further away from each other are less related than pixels that are closer together. In the SASA distance, the weights are defined as

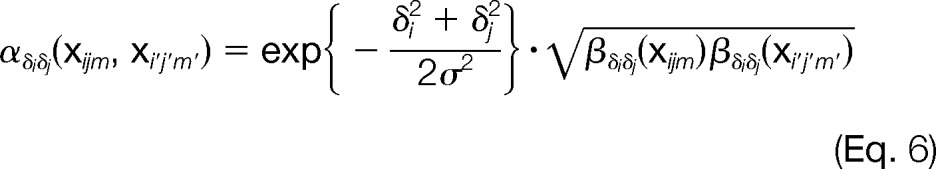
 where

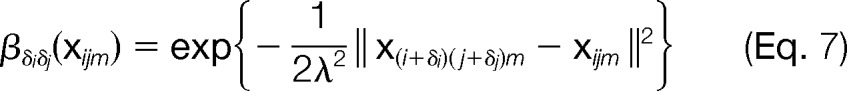
 which are adaptive weights that downweight neighborhood locations where the spectra are very different from the neighborhood center. This is designed to preserve edges between morphological regions and small details in local structure, which could otherwise be lost due to oversmoothing by the ordinary Gaussian weights. The term λ is set empirically to the half of the norm of the difference between the two most differing spectra in the neighborhood.

##### Defining the SA and SASA Distances to the Shrunken Centroid of a Class or Segment

The distance above can be adapted to express the distance between the individual pixels and the shrunken centroids as follows

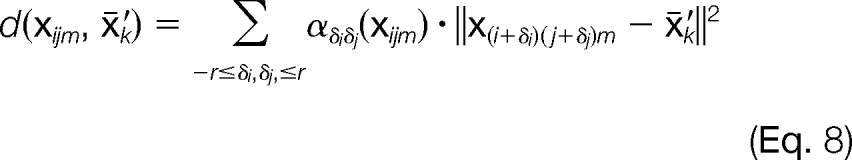
 where defining the α_δ*_i_*δ*_j_*_ using the Gaussian weights as in Equation 5 results in our version of the SA distance, and using adaptive weights defined as

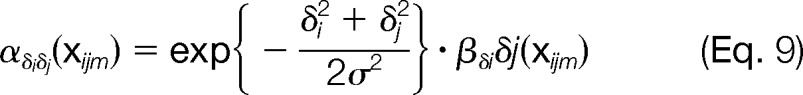
 with β_δ*_i_*δ*_j_*_(*x_ijm_*) as in Equation 7 results in our version of the SASA distance. We normalize the weights in both cases so that they sum to 1.

Unlike in Equation 4 above, in Equation 8, we consider the dissimilarity between a pixel's spectrum and a class (or segment), rather than the dissimilarity between the spectra at two pixels. Note that our version of the SASA distance has only one β_δ*_i_*δ*_j_*_ rather than two, reflecting this difference. In the case of supervised classification, we will use this distance to classify pixels according their spectrum's similarity to the shrunken centroids of known classes. In the case of unsupervised image segmentation, we will use this distance to iteratively update the pixels assigned to discovered segments.

Note also that, in both supervised and unsupervised situations, this requires the empirical selection of the shrinkage parameter *s*. Moreover, for unsupervised segmentation, the selection of the number of segments *K* is also required. The procedure for selecting these parameters and their effect and implications will be described further in Selection of Parameters.

##### Assignment of Segment or Class Probabilities to Pixels

For supervised classification nearest shrunken centroids can be interpreted as a regularized version of linear discriminant analysis (14, 15). In this case, each of the *K* classes has a prior probability π*_k_* and is modeled as a multivariate Gaussian distribution. All classes are assumed to share a common diagonal within-class covariance matrix. This leads to a straightforward way to calculate probabilities for individual observations belonging to a class using Gaussian likelihoods. By analogy, we calculate a discriminant score based on the SA or SASA distances from each spectrum x*_ijm_* to each of the shrunken centroids x̄*_k_* as

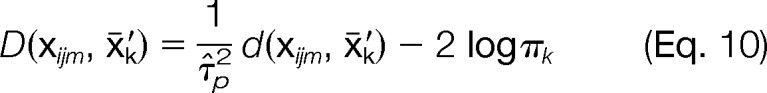
 where, as before, τ̂*_p_* is the pooled within-class standard deviation for feature *p*. We typically estimate the prior probabilities empirically as π̂*_k_* = *N_k_*/*N*. If the training data are not representative of the population, different priors could be used. Because we are using spatial distances that incorporate spectra from multiple pixels, the discriminant scores cannot be interpreted directly as following Gaussian distributions. However, we empirically demonstrate below that the technique still produces good results in practice. Therefore, we further follow Tibshirani et al. by calculating class probabilities for each spectrum x*_ijm_* for each class (or segment) *k* as

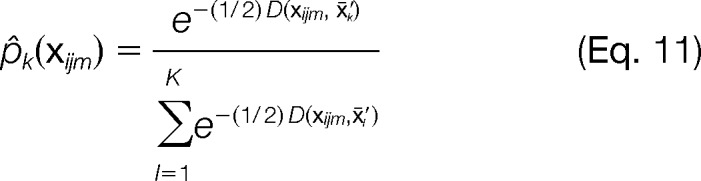


A pixel is assigned to the class with the highest *p̂_k_*(x*_ijm_*).

Unsupervised segmentation follows the same procedure. *K* is the maximum number of segments, and the segments are initialized randomly or with another segmentation procedure. We typically use π*_k_* = *1/K*, but a semi-supervised procedure could be developed that uses different priors and a different initialization procedure. During each iteration of the segmentation, a pixel is updated as belonging to the segment with the highest *p̂_k_*(x*_ijm_*) using Equation 11.

##### Selection of Parameters

The proposed framework requires the choice of the shrinkage parameter *s*, and, for unsupervised segmentation, the number of segments *K*.

In the case of supervised classification, the classes are known, and therefore, *s* can be selected by cross-validation. Specifically, given a set of *M* biological samples on *M* slides, each slide is viewed as an experimental unit for cross-validation. For experiments with a small number of biological replicates, *M*-fold (*i.e.* leave-one-sample-out) cross-validation can be performed. Within each fold (or sample) of cross-validation, fit spatial shrunken centroids for a range of values of *s*. The final selected values of *s* is the one that maximizes the overall classification accuracy on the left-out samples. This is illustrated for the human RCC experiment in Supplemental Fig. 10.

In the case of unsupervised segmentation, the individual segments and also the exact number of segments are unknown. However, there is a relationship between the number of informative features in the model, expressed by the shrinkage parameter *s*, and the number of segments *K*. First, spurious segments tend to be defined by noninformative features. When those are removed through statistical regularization, the spurious segments become empty. They have *N_k_* = 0, and are, in fact, removed. Second, excessive regularization can remove some informative features, and this results in the loss of the correct segments. We balance the regularization and the number of segments by creating segmentations for multiple values of *s* and *K* and then plotting the relationship between *s* and the number of nonempty segments. We illustrate this using experimental data in Section 3.4.2 and in Supplementary Section 2.2.2.

##### Algorithm and Implementation

The full algorithm for spatial shrunken centroids for a single set of parameters is described in Supplementary Section 2.1.1 for supervised classification, and in Supplementary Section 2.1.2 for unsupervised segmentation.

The proposed classification and segmentation methods are implemented in the R package Cardinal (cardinalmsi.org) (5), which is available from Bioconductor. Cardinal is free and open source and runs on Windows, Mac, and Linux.

**Fig. 3.**
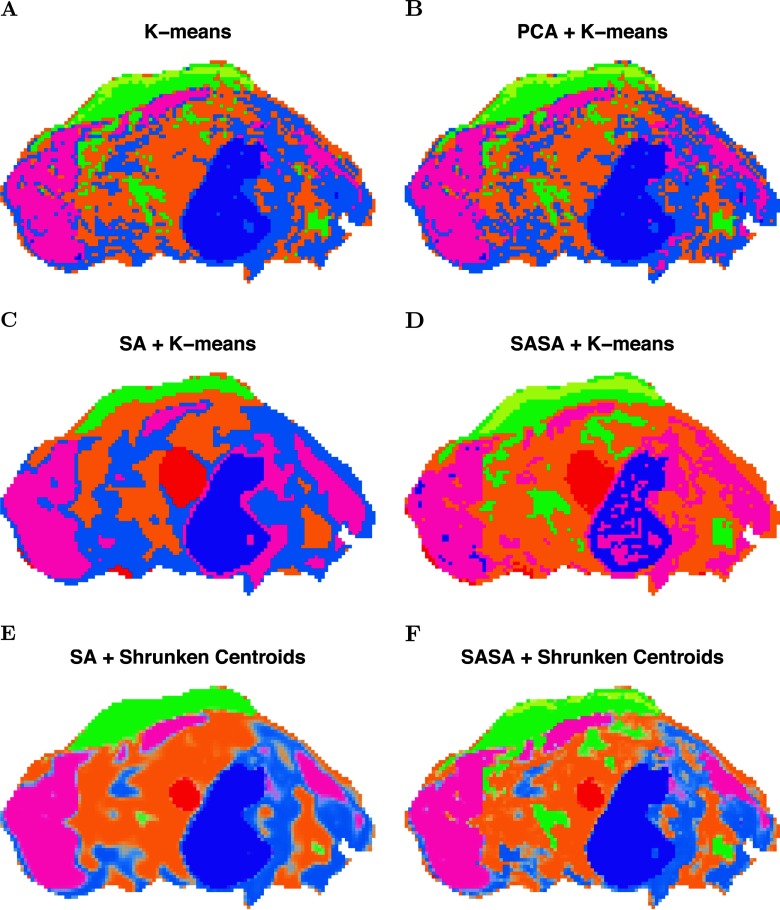
**Pig fetus cross-section: segmentation comparison.** (*A*) *K*-means clustering applied to the peak-picked spectra. (*B*) *K*-means clustering applied to the first five principal components of the peak-picked spectra. (*C*) Spatially aware (SA) clustering. (*D*) Spatially aware structurally adaptive (SASA) clustering. (*E*) Spatial shrunken centroids with SA distance. (*F*) Spatial shrunken centroids with SASA distance.

The implementation is efficient, utilizing C and C++ for speed. It can efficiently handle large datasets and is limited only by the requirement that the dataset must be fully loaded into memory. For example, the segmentations for the fetal pig dataset (143 peaks and 4,959 pixels) in Fig. 3*E* and Fig. 3*F* took 51 and 52 s, respectively. The segmentations for the cardinal painting (51 peaks and 12,600 pixels) in Supplemental Fig. 5*E* and Supplemental Fig. 5*F* took 67 and 48 s, respectively. The cross-validation for the human RCC dataset (850 features and 6,077 pixels) in Fig. 10 took 69 s.

##### Evaluation

##### Spatial Probabilistic Modeling Improves the Quality of Segmentation over Per-Pixel Segmentation

Spatial segmentations for the pig fetus cross-section dataset are illustrated in Fig. 3, which compares results from existing segmentation methods with the proposed segmentation method. In Fig. 3*A*, *k*-means clustering was applied to the peak-picked spectra, resulting in a noisy segmentation. The heart is not assigned to a unique segment. Figure 3*B* shows *k*-means clustering applied to the first five principal components of the peak-picked spectra, which also results in a noisy segmentation, again without the heart represented as a unique segment. Figure 3*C* and Fig. 3*D* show the spatially aware clustering and spatially aware structurally adaptive clustering of Alexandrov and Kobarg (4), which both result in cleaner segmentations with clearer edges between segments. The heart is assigned to a unique segment in both segmentations, as well as the brain and liver. All of the methods above require a predetermined number of segments, which was set to 6, based on the procedure described in the section Statistical Regularization Enables Data-Driven Selection of the Number of Segments for Unsupervised Experiments. Figure 3*E* and Fig. 3*F* show the proposed spatial shrunken centroids segmentation method with SA and SASA distances, which produce clean segmentations comparable to those in Fig. 3*C* and Fig. 3*D*. The number of segments for these methods was initialized to 20 and resulted in six segments in the final segmentations, as described in the section Statistical Regularization Enables Data-Driven Selection of the Number of Segments for Unsupervised Experiments. In addition, the segmentations in Fig. 3*E* and Fig. 3*F* are more similar to each other than those in Fig. 3*C* and Fig. 3*D*, suggesting that the proposed spatial shrunken centroids method produces more consistent results across different types of spatial smoothing.

Spatial segmentations of the cardinal painting are shown in Supplemental Fig. 5, which demonstrates the performance of existing and proposed methods compared with the ground truth.

##### Statistical Regularization Enables Data-Driven Selection of the Number of Segments for Unsupervised Experiments

**Fig. 4.**
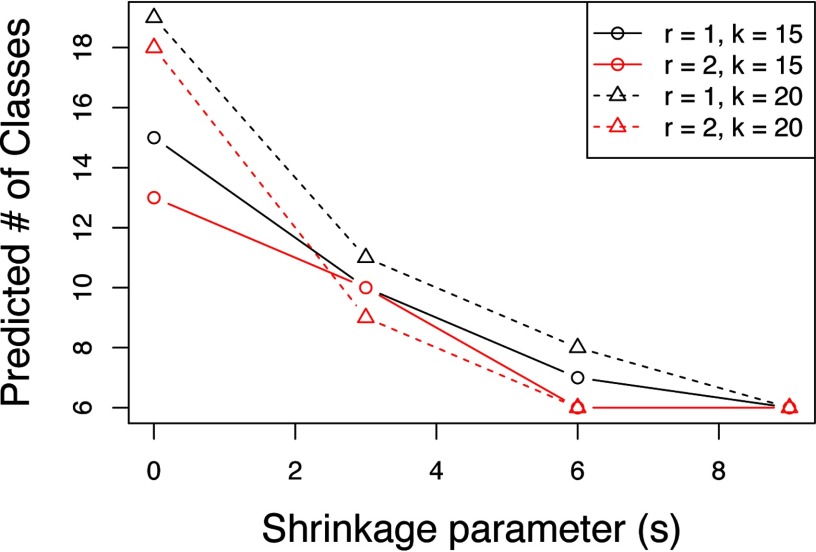
**Pig fetus cross-section: selection of the number of segments.** Spatial shrunken centroids with SA distance is performed with different shrinkage parameters (*s*), spatial smoothing radii (*r*), and starting numbers of segments (*K*).

The selection of the number of segments for the pig fetus cross-section segmentation from Fig. 3*E* is illustrated in Fig. 4. To select an appropriate segmentation, we initialize spatial shrunken centroids with different numbers of starting segments *K* (*e.g.* 15 and 20) while increasing the shrinkage parameter s (*e.g.* from 0 to 9 in increments of 3). Appropriate segmentations are those that result in a comparable number of predicted segments from different numbers of starting of segments. This suggests that all extraneous segments have been dropped, and the remaining segments explain true biology. For the pig fetus dataset in Fig. 4, this occurs after about *s* = 3. We also look for the predicted number of segments to stabilize (stop decreasing) as *s* increases. In Fig. 4, this occurs around *s* = 6. The segmentation in Fig. 3*E* above corresponds to *r* = 2, *K*= 20, *s* = 6.

Selection of the number of segments for the cardinal painting segmentation from Supplemental Fig. 5*E* is demonstrated in Supplemental Fig. 7.

##### Feature Selection Aids Interpretation by Automatically Selecting Spectral Features Associated with Differentiating Each Segment from Others

**Fig. 5.**
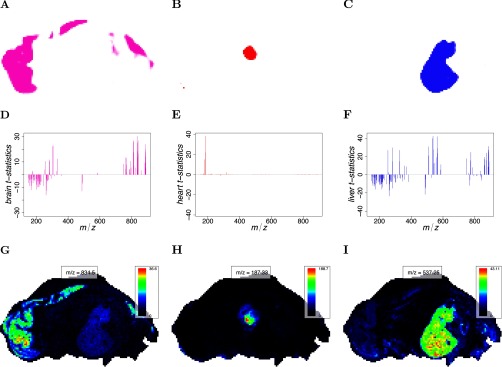
**Pig fetus cross-section: *t*-statistics and representative single ion images.** (*A–C*) The predicted segment membership probabilities from spatial shrunken centroids with SA distance. (*A*) the brain segment, (*B*) the heart segment, and (*C*) the liver segment. (*D–F*) The shrunken *t*-statistics of the spectral features. (*D*) The brain segment, (*E*) the heart segment, and (*F*) the liver segment. (*G–I*) The single ion images corresponding with the top-ranked spectral feature by shrunken *t*-statistic. (*G*) The brain segment, (*H*) the heart segment, and (*I*) the liver segment.

For the pig fetus cross-section segmentation from Fig. 3*E*, the selected spectral features using the proposed spatial shrunken centroids segmentation method are shown in Fig. 5. Feature selection is shown for the brain, heart, and liver segments, along with their corresponding *t*-statistics and top-ranked single ion images. Note that each unsupervised or supervised segment is characterized by its own reduced subset of informative features. Some features may be found informative for multiple segments, and some features may be found informative for no segment. For the brain segment, 49 spectral features were systematically enriched, and 54 features were systematically absent. For the heart segment, seven spectral features were systematically enriched, and one feature was systematically absent. For the liver segment, 41 spectral features were systematically enriched, and 74 features were systematically absent. Compared with the brain and liver segments, the heart had very few spectral features associated with it.

Feature selection for the cardinal painting segmentation from Supplemental Fig. 5E is shown in Supplemental Fig. 8.

##### Probabilistic Modeling Allows for Characterization and Visual Inspection of Uncertainty in Segment Membership in Unsupervised Experiments

**Fig. 6.**
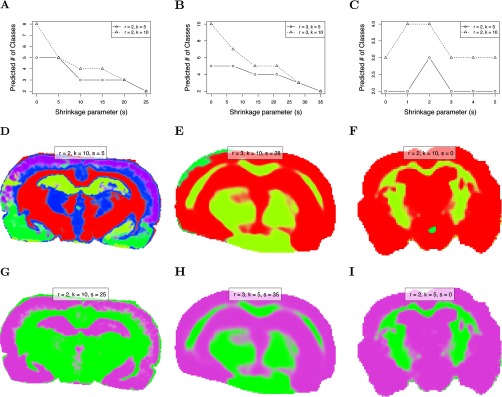
**Comparison of segmentation uncertainty in datasets of differing quality.** (*A–C*) show the predicted number of segments as the sparsity increases. (*A*) Rat brain (R1) with little noise. (*B*) Mouse brain (R2) with moderate noise. (*C*) Mouse brain (R3) with strong noise. (*D–F*) show the “best” segmentations selected by choosing the first (least sparse) segmentation after which the predicted number of segments are approximately equal for different initial numbers of segments. (*D*) Rat brain (R1). (*E*) Mouse brain (R2). (*F*) Mouse brain (R3). (*G-I*) show the segmentations resulting in two predicted segments through increasing sparsity. (*G*) Rat brain (R1). (*H*) Mouse brain (R2). (*I*) Mouse brain (R3).

The rodent brain datasets of varying quality were used to evaluate the ability of the proposed method to visually display uncertainty in its resulting segmentations. Because spatial shrunken centroids segmentation results in probabilities of segment membership, using transparency to reflect this probability creates a straightforward way of visually assessing uncertainty in a segmentation. Segmentations for the three rodent brain datasets are compared in Fig. 6.

Spatial shrunken centroids segmentation was performed for each rodent brain dataset with increasing shrinkage parameter (*s*), and the “best” segmentations were plotted in Figs. 6*D*-6*F* using the criteria described in section Statistical Regularization Enables Data-Driven Selection of the Number of Segments for Unsupervised Experiments for selecting an appropriate number of segments. This resulted in five segments for both the rat brain (R1) with little noise, three segments for the mouse brain (R2) with moderate noise, and three segments for the mouse brain (R3) with strong noise. For the strongly noisy mouse brain (R3), there was no clearly appropriate parameter set, as shown in Fig. 6*C*. Even for *K* = 10 starting segments, *s* = 0 resulted in only two predicted segments, and the predicted number of segments actually increased to three temporarily as *s* was increased before dropping to two again. This reflects the lower quality of the information in this brain dataset.

For the sake of comparison, the shrinkage parameter *s* was further increased past the point of stabilization until the predicted number of segments eventually dropped to only two segments. That is, more and more spectral features were excluded from the segmentation until the remaining ones only explained two segments. These segmentations are plotted in Figs. 6*G*–6*I*. For the rat brain (R1) with little noise, this occurred at *s* = 25. For the mouse brain (R2) with moderate noise, this occurred at *s* = 28. For the mouse brain (R3) with strong noise, this occurred at *s* = 0, reflecting the lesser amount of information in the data.

##### Classification in Supervised Experiments

**Fig. 7.**
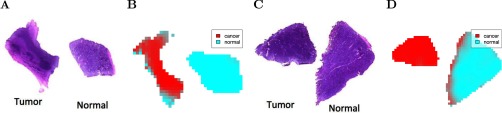
**Human renal cell carcinoma: classification.** For each matched pair, cancerous tissue is on the left, and healthy normal tissue is on the right. (*A*) Optical image of H&E-stained tissue pair UH0505_12. (*B*) Prediction based on spatial shrunken centroids for UH0505_12. (*C*) Optical image of H&E-stained tissue pair UH9812_03. (*D*) Prediction based on spatial shrunken centroids for UH9812_03.

Classification of the human RCC dataset using the proposed method is illustrated in Fig. 7 for two of the matched pairs. Eightfold cross-validation was used to select the shrinkage parameter, as illustrated in Supplementary Fig. 10. Spatial shrunken centroids achieves 88.9% cross-validated accuracy, defined as correctly classifying pixels as cancer or normal with respect to the manual annotation of the entire tissue. By comparison, PLS-DA applied to the same dataset achieves 96.8% cross-validated accuracy, and O-PLS-DA achieves 95.4%

**Fig. 8.**
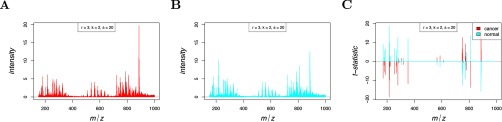
**Human renal cell carcinoma: shrunken centroids and *t*-statistics.** (*A*) The shrunken centroids for cancerous tissue. (*B*) The shrunken centroids for normal tissue. (*C*) The shrunken *t*-statistics for normal and cancerous tissue, showing 215 *m/z* (*t'_normal_*_,215_ = 18.83) is strongly associated with normal tissue, and 886 *m/z* (*t'_cancer_*_,886_ = 15.9) is strongly associated with cancerous tissue.

A clear advantage of spatial shrunken centroids for classification is its selection of informative features that differentiate each class, as shown in Fig. 8*C*. Unlike PLS-DA and O-PLS-DA, which use all features, making interpretation difficult, spatial shrunken centroids only uses the features that best distinguish each class. Among the selected features, the top ion associated with cancerous tissue was 885.7 *m/z*, which is known to be more abundant in cancer (8). The top ion associated with normal tissue was 215.3 *m/z*, which is known to be more abundant in normal tissue (8).

Another advantage of spatial shrunken centroids is the estimation of probabilities of class membership. Plotting these probabilities with transparency allows visual assessment of the confidence in the prediction. This can help pinpoint heterogeneous regions of the individual tissues, and possible inconsistencies in manual whole-tissue annotations. For example, in Fig. 7*B*, the tumor tissue (*left*) shows an indistinct border of normal tissue along the left side, and in Fig. 7*D*, the normal tissue (*right*) shows an indistinct border of tumor tissue along the left side. These borders are defined by ions known to be associated with cancer and normal tissue (8) (Supplemental Fig. 3 and Supplemental Fig. 4). Therefore, the manual annotation may be imprecise, and PLS-DA and O-PLS-DA may be overfitting.

## DISCUSSION

The manuscript introduced a general statistical framework, called spatial shrunken centroids, for both unsupervised segmentation and supervised classification of MS imaging experiments. For unsupervised segmentation, it produces better segmentations than *k*-means clustering of the mass spectra or *k*-means clustering of their principal components. It outputs probabilities of segment membership and therefore helps characterize and visualize the uncertainty in the segmentation. It automatically selects the total number of segments, as well as subsets of informative features that define each segment to provide more interpretable results. For supervised classification, spatial shrunken centroids achieves similar accuracy as compared with commonly used methods such as PLS-DA and O-PLS-DA. However, similarly to the unsupervised segmentation, it characterizes and visualizes the uncertainty of segment membership and subsets of informative features that define each class.

Spatial shrunken centroids is designed to work with data obtained after signal processing. It takes as input a set of previously detected, quantified, aligned, and normalized features and is not designed for detecting such features from the raw data anew. Also, spatial shrunken centroids does not require a previous identification on the underlying analytes. The approach only aims at interpreting the quantitative information in the spectra, and this can be done with or without the knowledge of the analyte identity. However, spatial shrunken centroids can potentially enhance the process of identification. For example, the informative subsets of features selected in each segment or class can reduce the possible search space of analytes that we would like to identify.

This framework has been previously implemented in the open-source R package Cardinal (5). The implementation in Cardinal can efficiently handle large datasets, as long as they can be loaded into memory and can be used as part of larger MS imaging data processing pipelines. We hope that the flexibility, versatility, and efficiency of the method will make it a useful tool for biological and clinical investigations.

## Supplementary Material

Supplemental Data

## Footnotes

^1^ The abbreviations used are:

DESI: desorption electrospray ionization
MALDI: matrix-assisted laser desorption/ionization
MS: mass spectrometry
*m/z*: mass-to-charge ratio
O-PLS-DA: orthogonal projection to latent structures discriminant analysis
PLS-DA: projection to latent structures discriminant analysis
RCC: renal cell carcinoma
SA: spatially aware
SASA: spatially aware structurally adaptive
TOF: time-of-flight.

## References

[1] Alexandrov T. (2012) MALDI imaging mass spectrometry: Statistical data analysis and current computational challenges. BMC Bioinformatics 13, S11 23176142 10.1186/1471-2105-13-S16-S11 PMC3489526

[2] Alexandrov T., and Bartels A. (2013) Testing for presence of known and unknown molecules in imaging mass spectrometry. Bioinformatics 29, 2335–2342 23873892 10.1093/bioinformatics/btt388

[3] Alexandrov T., Becker M., Deininger S. O., Ernst G., Wehder L. Grasmair M., von Eggeling F., Thiele H., and Maass P. (2010) Spatial segmentation of imaging mass spectrometry data with edge-preserving image denoising and clustering. J. Proteome Res. 9, 6535–6546 20954702 10.1021/pr100734z

[4] Alexandrov T., and Kobarg J. H. (2011) Efficient spatial segmentation of large imaging mass spectrometry datasets with spatially aware clustering. Bioinformatics 27, i230–8 21685075 10.1093/bioinformatics/btr246 PMC3117346

[5] Bemis K. D., Harry A., Eberlin L. S., Ferreira C., van de Ven S. M., Mallick P., Stolowitz M., and Vitek O. (2015) Cardinal: An R package for statistical analysis of mass spectrometry-based imaging experiments. Bioinformatics10.1093/bioinformatics/btv146 PMC4495298 25777525

[6] Deininger S. O., Ebert M. P., Fütterer A., Gerhard M., and Röcken C. (2008) MALDI imaging combined with hierarchical clustering as a new tool for the interpretation of complex human cancers. J. Proteome Res. 7, 5230–5236 19367705 10.1021/pr8005777

[7] Dill A. L., Eberlin L. S., Costa A. B., Zheng C., Ifa D. R., Cheng L., Masterson T. A., Koch M. O., Vitek O., and Cooks R. G. (2011) Multivariate statistical identification of human bladder carcinomas using ambient ionization imaging mass spectrometry. Chemistry 17, 2897–2902 21284043 10.1002/chem.201001692 PMC3050580

[8] Dill A. L., Eberlin L. S., Zheng C., Costa A. B., Ifa D. R., Cheng L., Masterson T. A., Koch M. O., Vitek O., and Cooks R. G. (2010) Multivariate statistical differentiation of renal cell carcinomas based on lipidomic analysis by ambient ionization imaging mass spectrometry. Anal. Bioanal. Chem. 398, 2969–2978 20953777 10.1007/s00216-010-4259-6 PMC10712022

[9] Eberlin L. S., Tibshirani R. J., Zhang J., Longacre T. A., Berry G. J., Bingham D. B., Norton J. A., Zare R. N., and Poultsides G. A. (2014) Molecular assessment of surgical-resection margins of gastric cancer by mass-spectrometric imaging. Proc. Natl. Acad. Sci. U.S.A. 111, 2436–2441 24550265 10.1073/pnas.1400274111 PMC3932851

[10] Ferguson L. S., Wulfert F., Wolstenholme R., Fonville J. M., Clench M. R., Carolan V. A., and Francese S. (2012) Direct detection of peptides and small proteins in fingermarks and determination of sex by MALDI mass spectrometry profiling. Analyst 137, 4686–4692 22950080 10.1039/c2an36074h

[11] Macini N. E., Eijkel G. B., ter Brugge P., Jonkers J. Wesseling J., and Heeren R. M. A. (2015) The Use of Mass Spectrometry Imaging to Predict Treatment Response of Patient-Derived Xenograft Models of Triple-Negative Breast Cancer. J. Proteome Res. 14, 1069–1075 25553735 10.1021/pr501067z

[12] McCombie G., Staab D., Stoeckli M., and Knochenmuss R. (2005) Spatial and spectral correlations in MALDI mass spectrometry images by clustering and multivariate analysis. Anal. Chem. 77, 6118–6124 16194068 10.1021/ac051081q

[13] Sarkari S., Kaddi C. D., Bennett R. V., Fernández F. M., and Wang M. D. (2014) Comparison of clustering pipelines for the analysis of mass spectrometry imaging data 10.1109/EMBC.2014.6944691 25571059

[14] Tibshirani R., Hastie T., Narasimhan B., and Chu G. (2002) Diagnosis of multiple cancer types by shrunken centroids of gene expression. Proc. Natl. Acad. Sci. U.S.A. 99, 6567–6572 12011421 10.1073/pnas.082099299 PMC124443

[15] Tibshirani R., Hastie T., Narasimhan B., and Chu G. (2003) Class prediction by nearest shrunken with applications to DNA microarrays. Statistical Science 18, 104–117

[16] Veselkov K. A., Mirnezami R., Strittmatter N., Goldin R. D., Kinross J., Speller A. V., Abramov T., Jones E. A., Darzi A., Holmes E., Nicholson J. K., and Takats Z. (2014) Chemo- informatic strategy for imaging mass spectrometry-based hyperspectral profiling of lipid signatures in colorectal cancer. Proc. Natl. Acad. Sci. U.S.A. 111, 1216–1221 24398526 10.1073/pnas.1310524111 PMC3903245

[17] Watrous J. D., Alexandrov T., and Dorrestein P. C. (2011) The evolving field of imaging mass spectrometry and its impact on future biological research. J. Mass Spectrom. 46, 209–222 21322093 10.1002/jms.1876 PMC3303182

[18] Wijetunge C. D., Saeed I., Boughton B. A., Spraggins J. M., Caprioli R. M., Bacic A., Roessner U., and Halgamuge S. K. (2015) EXIMS: An improved data analysis pipeline based on a new peak picking method for exploring imaging mass spectrometry data. Bioinformatics 31, 3198–3206 26063840 10.1093/bioinformatics/btv356

[19] Wiseman J. M., and Laughlin B. C. 2007) Desorption electrospray ionization (DESI) mass spectrometry: A brief introduction and overview. Current Separations Drug Development 22, 11–14

[20] Wu C., Dill A. L., Eberlin L. S., Cooks R. G., and Ifa D. R. (2013) Mass spectrometry imaging under ambient conditions. Mass Spectrom. Rev. 32, 218–243 22996621 10.1002/mas.21360 PMC3530640

[21] Yang C., He Z., and Yu W. (2009) Comparison of public peak detection algorithms for MALDI mass spectrometry data analysis. BMC Bioinformatics 10, 4 19126200 10.1186/1471-2105-10-4 PMC2631518

