# Supplementary material for: Probabilistic Segmentation of Mass Spectrometry (MS) Images Helps Select Important Ions and Characterize Confidence in the Resulting Segments
Source: Mol Cell Proteomics. 2016 Jan 21;15(5):1761–72. doi: 10.1074/mcp.O115.053918 (PMC4858953; doi:10.1074/mcp.O115.053918)
Supplement: Supplemental Data [file supp_15_5_1761__index.html]

Probabilistic segmentation of mass spectrometry images helps select important ions and characterize confidence in the resulting segments — Probabilistic Segmentation of Mass Spectrometry (MS) Images Helps Select Important Ions and Characterize Confidence in the Resulting Segments — Probabilistic Segmentation of Mass Spectrometry Images — Supplemental Data 

# Probabilistic Segmentation of Mass Spectrometry (MS) Images Helps Select Important Ions and Characterize Confidence in the Resulting Segments

## Supplemental Data

- Supplementary Materials (.pdf, 4.7 MB) - Supplementary Material
