# Supplementary material for: Probabilistic Segmentation of Mass Spectrometry (MS) Images Helps Select Important Ions and Characterize Confidence in the Resulting Segments
Source: Mol Cell Proteomics. 2016 Jan 21;15(5):1761–72. doi: 10.1074/mcp.O115.053918 (PMC4858953; doi:10.1074/mcp.O115.053918)
Supplement: Supplemental Data [file 10.1074_O115.053918_mcp.O115.053918-1.pdf]

Supplementary Materials

Probabilistic segmentation of mass spectrometry images helps select important ions and characterize confidence in the resulting segments

July 20, 2015

Kyle D. Bemis [1], April Harry[1], Livia S. Eberlin[2], Christina R. Ferreira[2], Stephanie M. van de Ven[3], Parag Mallick[3], Mark Stolowitz[3], Olga Vitek [4,5]

[1]Department of Statistics, Purdue University, West Lafayette, IN USA 47907

[2]Department of Chemistry, Purdue University, West Lafayette, IN USA 47907

[3]Canary Center at Stanford for Cancer Early Detection, Stanford University School of Medicine, Palo Alto, CA USA 94304

[4]College of Science, Northeastern University, Boston, MA USA 02115

[5]College of Computer and Information Science, Northeastern University, Boston, MA USA 02115

Contents

1 Experimental Procedures **3**

1.1 Unsupervised segmentation: cardinal painting with known segmentation 3

1.2 Supervised segmentation: human renal cell carcinoma 4

2 Results **7**

2.1 Algorithm and implementation 7

2.1.1 Procedure for spatial shrunken centroids classification (supervised) 7

2.1.2 Procedure for spatial shrunken centroids segmentation (unsupervised) 8

2.2 Evaluation 10

2.2.1 Spatial probabilistic modeling provides better quality segmentation as compared to per-pixel segmentation 10

2.2.2 Statistical regularization enables data-driven selection of the number of segments for unsupervised experiments 12

2.2.3 Feature selection aids interpretation by automatically identifying spectral features associated with differentiating each segment from others 15

2.2.4 Classification in supervised experiments 17

3 References **19**

1 Experimental Procedures

1.1 Unsupervised segmentation: cardinal painting with known segmentation

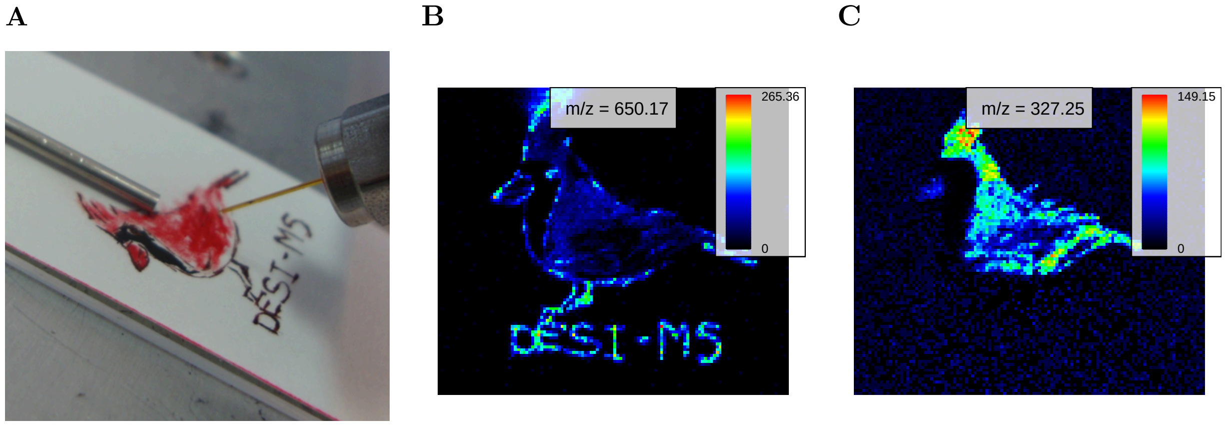

Supplementary Figure 1: **Cardinal painting: optical image and single ion images.** *A*, Optical image of the cardinal painting during collection of mass spectra. *B–C*, characteristic single ion images for the cardinal painting dataset at *B*, 650.17 m/z, showing the “DESI-MS” text, and *C*, 327.25 m/z, showing the body (red pigment).

1.2 Supervised segmentation: human renal cell carcinoma

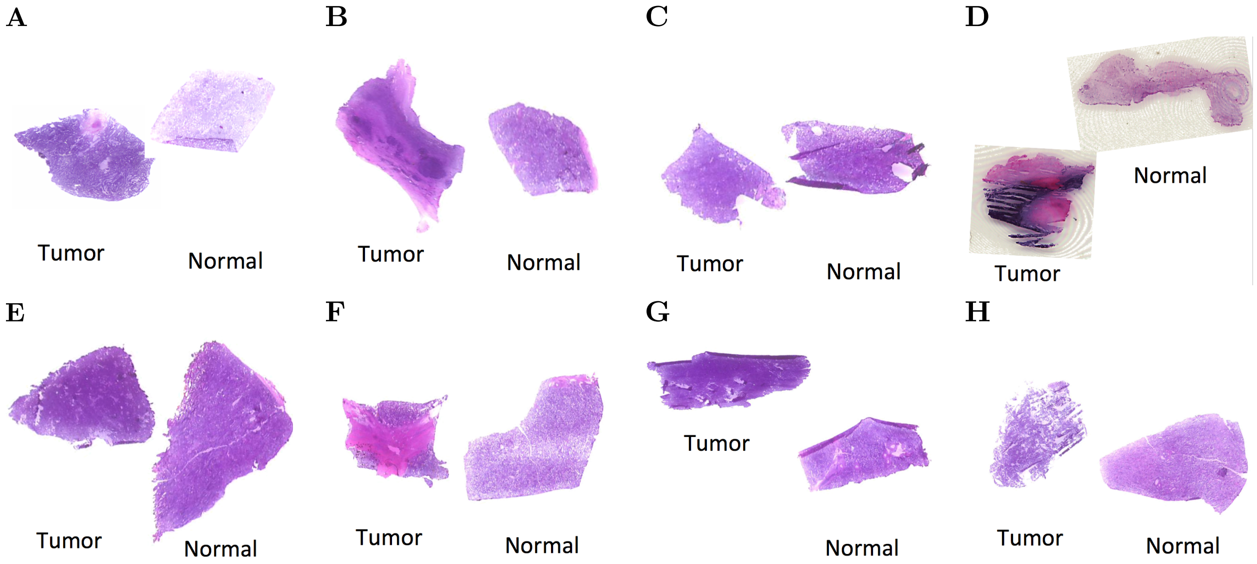

Supplementary Figure 2: **Human renal cell carcinoma: morphology.** The morphology is characterized by optical images of the H&E stained tissues. For each matched pair, cancerous tissue is on the left, and normal tissue is on the right.

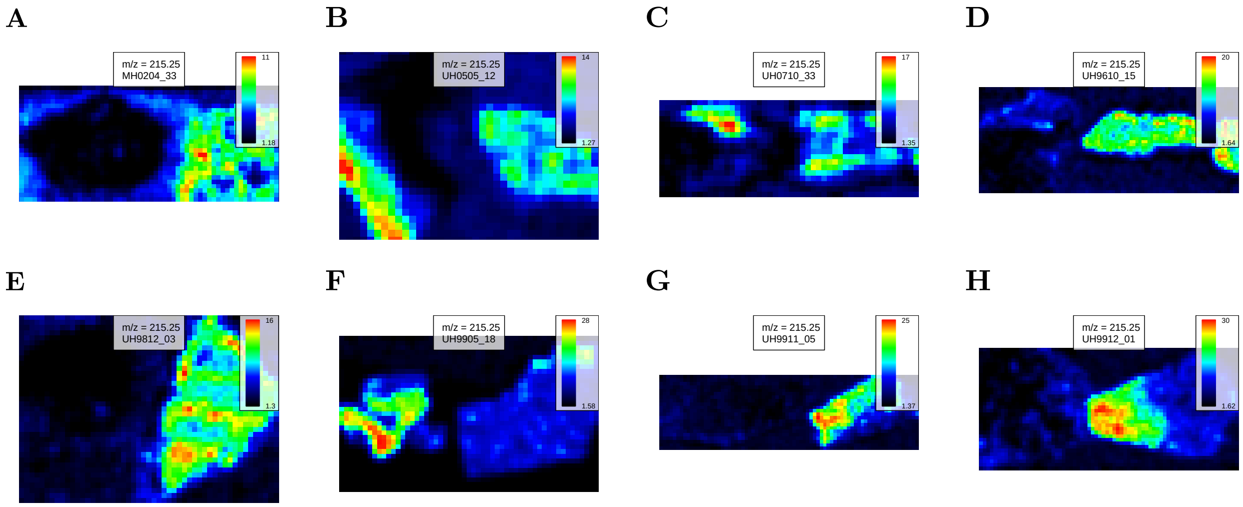

Supplementary Figure 3: **Human renal cell carcinoma: normal tissue single ion images.** For each matched pair, cancerous tissue is on the left, and normal tissue is on the right. 215.25 m/z is known to be more abundant in normal tissue [3]. Note that some of the cancerous tissues appear to have regions of normal tissue, such as samples B, UH0505_12, C, UH0710_33, and F, UH9905_18.

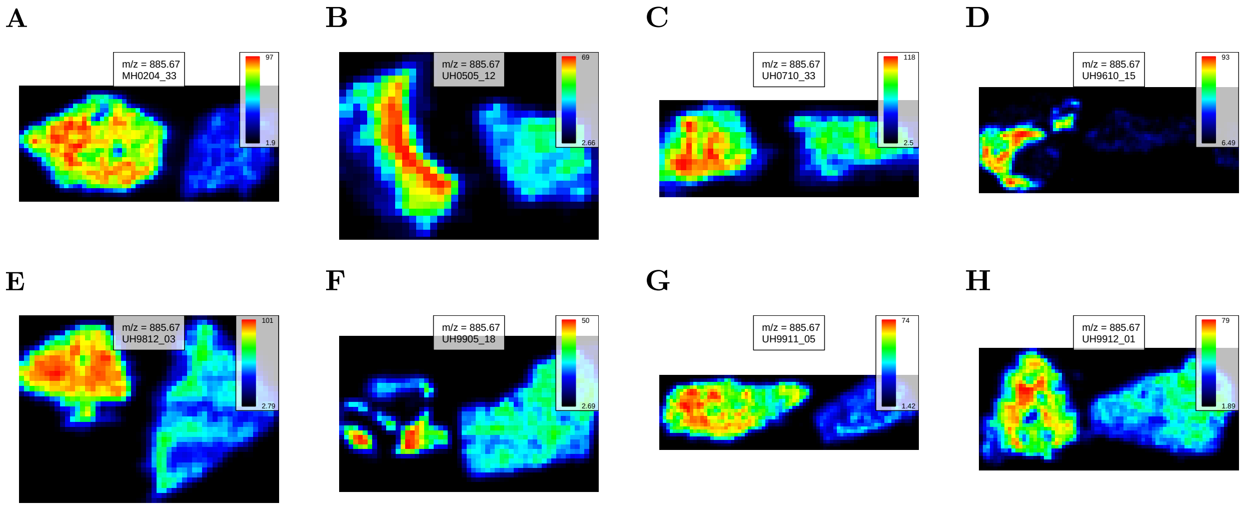

Supplementary Figure 4: **Human renal cell carcinoma: cancer tissue single ion images.** For each matched pair, cancerous tissue is on the left, and normal tissue is on the right. 885.67 m/z is known to be more abundant in cancerous tissue [3]. Note that some of the normal tissues appear to have regions of cancerous tissue, such as the left edge of sample E, UH9812_03.

2 Results

2.1 Algorithm and implementation

All the referenced equations can be found in the main text. Both algorithms are implemented in the R package **Cardinal** (`cardinalmsi.org`) [2], available from Bioconductor. Source code is available at `http://github.com/kbemis/Cardinal`.

2.1.1 Procedure for spatial shrunken centroids classification (supervised)

The following describes the algorithm for a single set of parameters for a single fold of cross-validation. Parameters should be selected as described in Section 5.2.6.

Input

1. Training set of samples with class labels $k = 1, \ldots, K$
2. Testing set of samples $m = 1, \ldots, M$
3. Parameters
   (a) Neighborhood radius r
   (b) Shrinkage parameter s

Fitting – performed on samples from the training set

1. Calculate the overall centroid $\bar{\mathbf{x}}$
2. For each class $k = 1, \ldots, K$:
   (a) Calculate the class centroid $\bar{\mathbf{x}}_k$
   (b) For each feature $p = 1, \ldots, P$:
      i. Calculate the t-statistics t_{kp} [Equation 1].
      ii. Calculate the shrunken t-statistics t'_{kp} [Equation 2].
   (c) Calculate the class shrunken centroid $\bar{\mathbf{x}}'_k$ [Equation 3].

3. Output the shrunken t-statistics t'_{kp} and shrunken centroids $\bar{\mathbf{x}}'_k$.

Prediction – performed on samples from the testing set

1. For each pixel at a location (i, j) on sample $m = 1, \ldots, M$:

   (a) For each class $k = 1, \ldots, K$:

      i. Calculate the distance to the class centroid $d(\mathbf{x}_{ijm},\ \mathbf{x}'_k)$ [Equation 8].

      ii. Calculate discriminant score $\mathcal{D}(\mathbf{x}_{ijm},\ \bar{\mathbf{x}}'_k)$ [Equation 10].

      iii. Calculate the class membership probability $\hat{p}_k(\mathbf{x}_{ijm})$ [Equation 11]

   (b) Assign the pixel to the class with the highest probability $\hat{p}_k(\mathbf{x}_{ijm})$.

2. Output the class assignments and class probabilities $\hat{p}_k(\mathbf{x}_{ijm})$.

2.1.2 Procedure for spatial shrunken centroids segmentation (unsupervised)

The following describes the algorithm for a single set of parameters. Parameters should be selected as described in Section 5.2.6.

Input

1. Unlabeled samples $m = 1, \ldots, M$

2. Maximum number of iterations *iter.max*

3. Parameters

   (a) Neighborhood radius r

   (b) Maximum number of segments K

   (c) Shrinkage parameter s

Fitting

1. Use SA or SASA clustering by Alexandrov and Kobarg [1] to initialize the K segments.

2. Calculate the overall centroid $\bar{\mathbf{x}}$

3. For each segment $k = 1, \ldots, K$ with $N_k \neq 0$:

   (a) Calculate the segment centroid $\bar{\mathbf{x}}_k$

   (b) For each feature $p = 1, \ldots, P$:

      i. Calculate the t-statistics t_{kp} [Equation 1].

      ii. Calculate the shrunken t-statistics t'_{kp} [Equation 2].

   (c) Calculate the segment shrunken centroid $\bar{\mathbf{x}}'_k$ [Equation 3].

4. For each pixel at a location (i, j) on sample $m = 1, \ldots, M$:

   (a) For each segment $k = 1, \ldots, K$:

      i. Calculate the distance to the segment shrunken centroid $d(\mathbf{x}_{ijm},\ \mathbf{x}'_k)$ [Equation 8].

      ii. If a segment has $N_k = 0$, define the distance to it as $d(\mathbf{x}_{ijm},\ \mathbf{x}'_k) = \infty$.

      iii. Calculate discriminant score $\mathcal{D}(\mathbf{x}_{ijm},\ \bar{\mathbf{x}}'_k)$ [Equation 10].

      iv. Calculate the segment membership probability $\hat{p}_k(\mathbf{x}_{ijm})$ [Equation 11]

   (b) Assign the pixel to the segment with the highest probability $\hat{p}_k(\mathbf{x}_{ijm})$.

5. Update the segments with the pixel assignments from step 4b.

6. Repeat steps 3–5 until no segments change, or at most *iter.max* times.

7. Output the shrunken t-statistics t'_{kp}, shrunken centroids $\bar{\mathbf{x}}'_k$, and probabilities $\hat{p}_k(\mathbf{x}_{ijm})$.

2.2 Evaluation

2.2.1 Spatial probabilistic modeling provides better quality segmentation as compared to per-pixel segmentation

Spatial segmentations of the cardinal painting are illustrated in Supplementary Figure 5, which demonstrates the performance of existing and proposed methods compared to the ground truth.. In Supplementary Figure 5A, k-means clustering was applied to the peak-picked spectra, resulting in a noisy segmentation. The face (black feathers) are not represented as a unique segment. Supplementary Figure 5B shows k-means clustering applied to the first five principal components of the peak-picked spectra, which also results in a noisy segmentation, but with all parts of the painting represented as segments. Supplementary Figure 5C and Supplementary Figure 5D show the spatially-aware clustering and spatially-aware structurally-adaptive clustering of Alexandrov and Kobarg [1], which both result in cleaner segmentations with clearer edges between segments. The methods above, which require a predetermined number of segments, were set to 8 segments. Supplementary Figure 5E and Supplementary Figure 5F show the proposed spatial shrunken centroids segmentation method with SA and SASA distances, which produce clean segmentations comparable for Supplementary Figure 5C and Supplementary Figure 5D. The proposed method was initialized to 10 segments, resulting in 8 segments in the final segmentations.

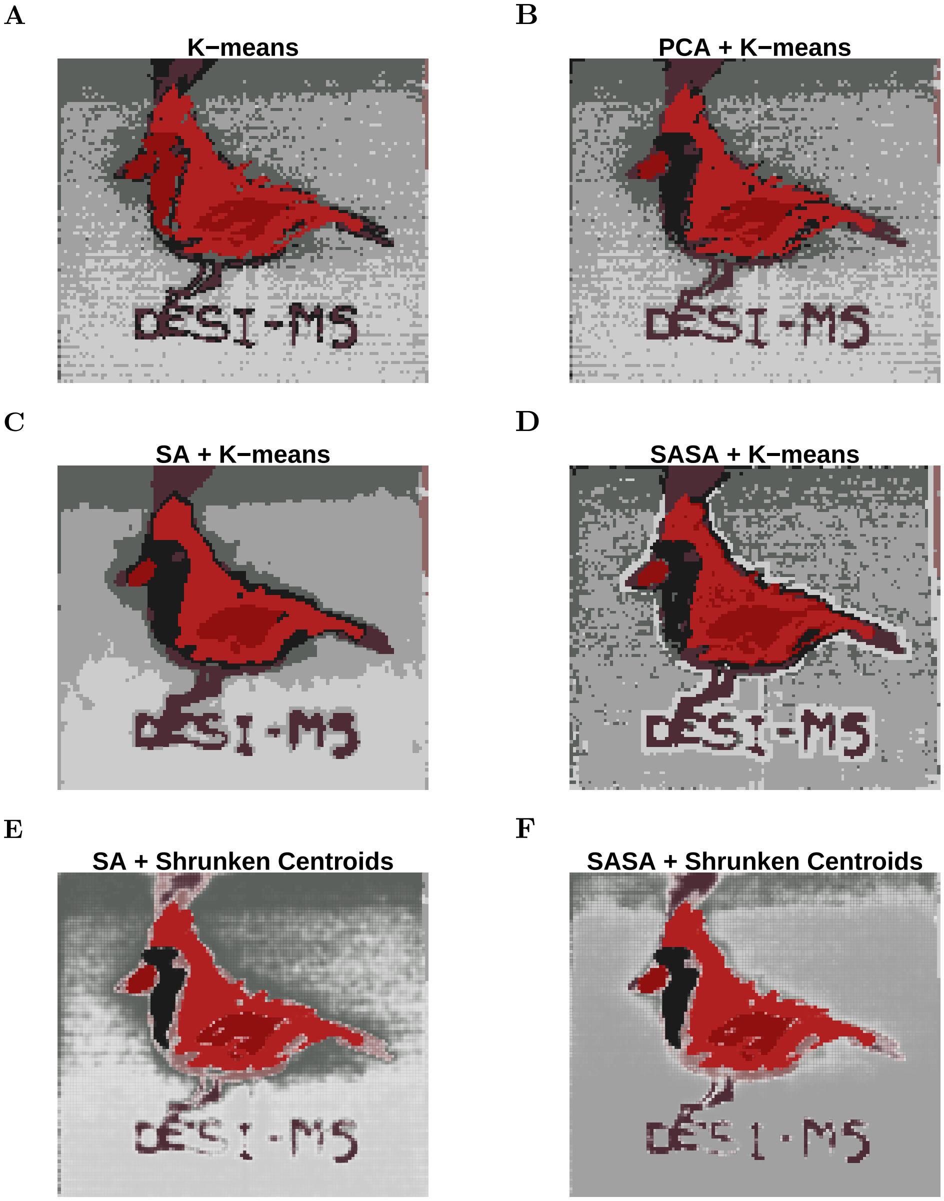

Supplementary Figure 5: **Cardinal painting: segmentation comparison.** *A*, K-means clustering applied to the peak-picked spectra. *B*, K-means clustering applied to the first five principal components of the peak-picked spectra. *C*, Spatially-aware (SA) clustering. *D*, Spatially-aware structurally-adaptive (SASA) clustering. *E*, Spatial shrunken centroids with SA distance. *F*, Spatial shrunken centroids with SASA.

2.2.2 Statistical regularization enables data-driven selection of the number of segments for unsupervised experiments

The selection of the number of segments for the pig fetus cross-section dataset is illustrated in Supplementary Figure 6. Supplementary Figure 6A shows the predicted number of segments for increasing shrinkage parameter s for spatial shrunken centroids with the spatially-aware (SA) distance. Supplementary Figure 6B shows the same for spatial shrunken centroids with the spatially-aware structurally-adaptive (SASA) distance. The method was initialized for spatial smoothing radii $r = 1$ and $r = 2$, and for starting number of segments $K = 15$ and $K = 20$. The shrinkage parameter s was increased from 0 to 9 in increments of 3.

To identify segmentations with the most appropriate number of segments, we first look for where the predicted number of segments become similar across different numbers of starting segments K. When this happens, only meaningful segments should remain. This occurs around $s = 3$. Next, we look for where the predicted number of segments stabilizes, which should correspond with an "elbow" in the graph, similar to a scree plot. For Supplementary Figure 6A, this occurs at $s = 6$, but for Supplementary Figure 6B, this may occur earlier at Supplementary Figure 6B $s = 3$.

Supplementary Figure 6C–F show the segmentions resulting for increasing shrinkage parameter s for spatial smoothing radius $r = 2$ and starting number of segments $K = 20$.

The selection of the number of segments for the cardinal painting dataset is illustrated in Supplementary Figure 7. Supplementary Figure 7A shows the predicted number of segments for increasing shrinkage parameter s for spatial shrunken centroids with SA distance. Supplementary Figure 7B shows the same for spatial shrunken centroids with SASA distance. The method was initialized for spatial smoothing radii $r = 1$ and $r = 2$, and for starting number of segments $K = 10$ and $K = 15$. The shrinkage parameter s was increased from 0 to 9 in increments of 3. For both versions, the segmentations begin to stabilize around $s = 3$.

Supplementary Figure 7C–F show the segmentions resulting for increasing shrinkage parameter s for spatial smoothing radius $r = 2$ and starting number of segments $K = 15$.

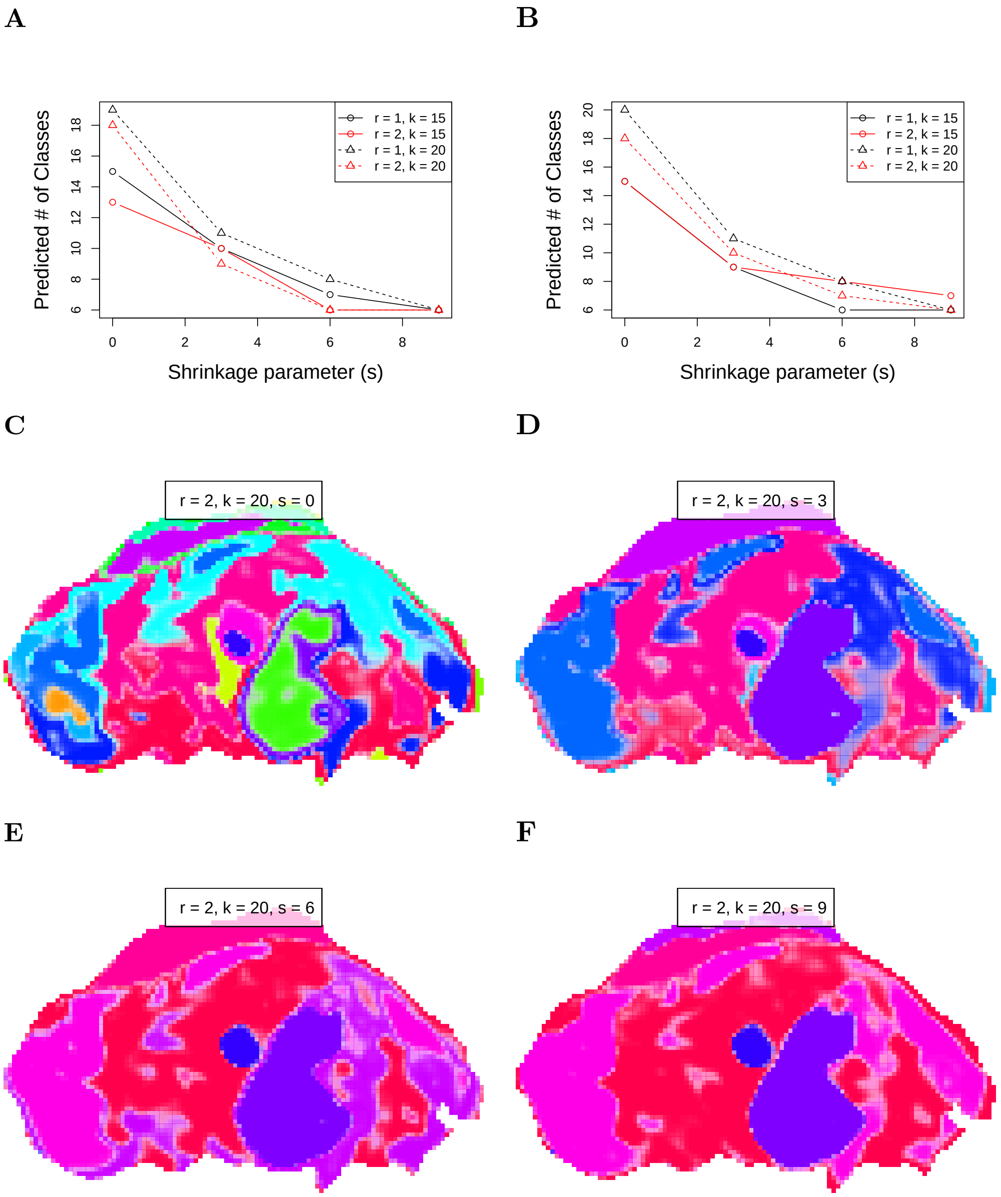

Supplementary Figure 6: **Pig fetus cross-section: selection of the number of segments.** *A*, Spatially-aware (SA) distance. *B*, Spatially-aware structurally-adaptive (SASA) distance. *C–F*, Segmentations using SA distance with smoothing radius of 2 and 20 initial segments, for increasing sparsity parameter s. *C*, $s = 0$, *D*, $s = 3$, *E*, $s = 6$, *F*, $s = 9$.

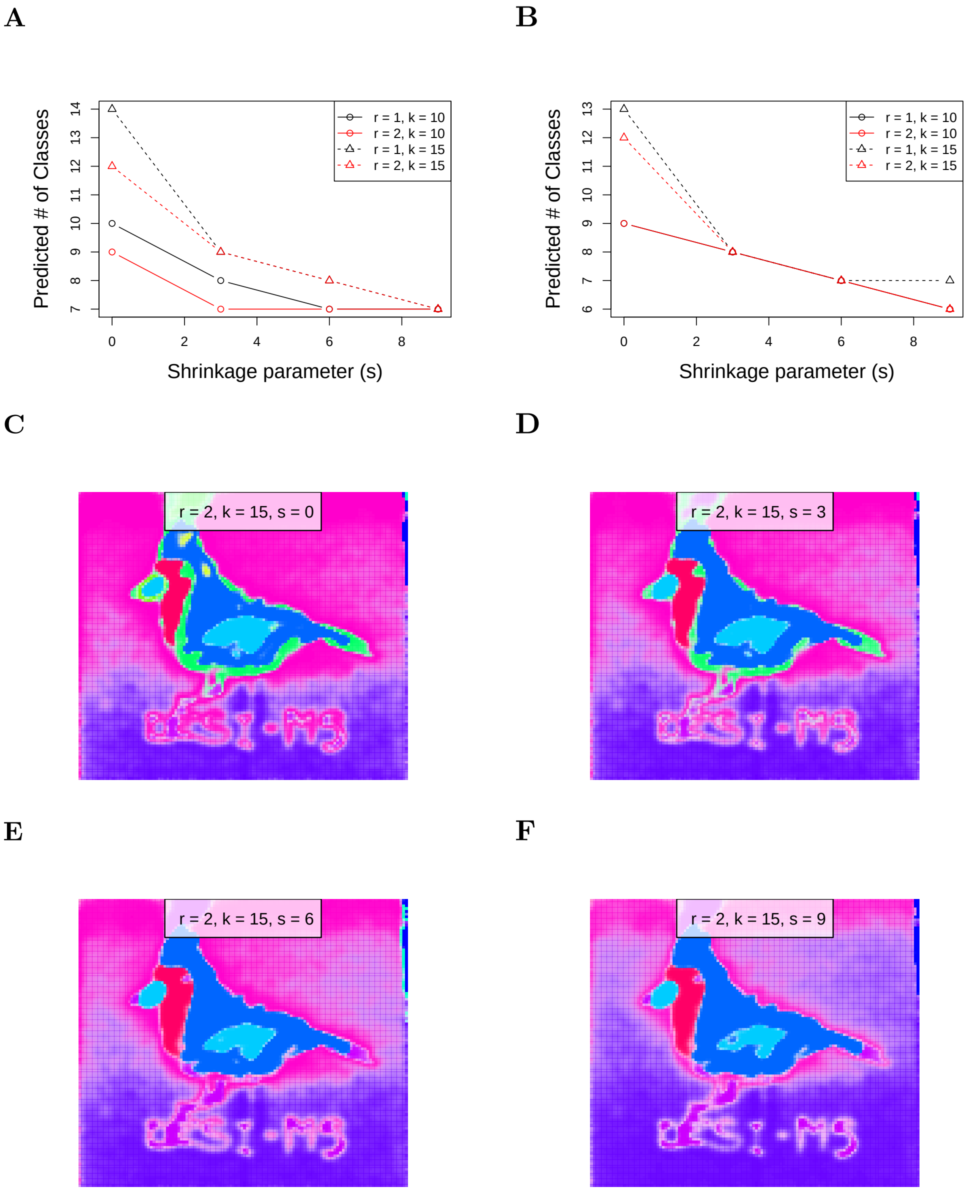

Supplementary Figure 7: **Cardinal painting: selection of the number of segments.** *A*, Spatially-aware (SA) distance. *B*, Spatially-aware structurally-adaptive (SASA) distance. *C*–*F*, Segmentations using SA distance with smoothing radius of 2 and 20 initial segments, for increasing sparsity parameter s. *C*, $s = 0$, *D*, $s = 3$, *E*, $s = 6$, *F*, $s = 9$.

2.2.3 Feature selection aids interpretation by automatically identifying spectral features associated with differentiating each segment from others

For the cardinal painting segmentation from Supplementary Figure 5E, the selected spectral features using the proposed spatial shrunken centroids segmentation method are shown in Supplementary Figure 8. Feature selection is shown for the text, body, and wing segments, along with their corresponding t-statistics and top-ranked single ion images. For the text segment, 4 spectral features were systematically enriched, and 27 features were systematically absent. For the body segment, 12 spectral features were systematically enriched, and 36 features were systematically absent. For the wing segment, 13 spectral features were systematically enriched, and 20 features were systematically absent.

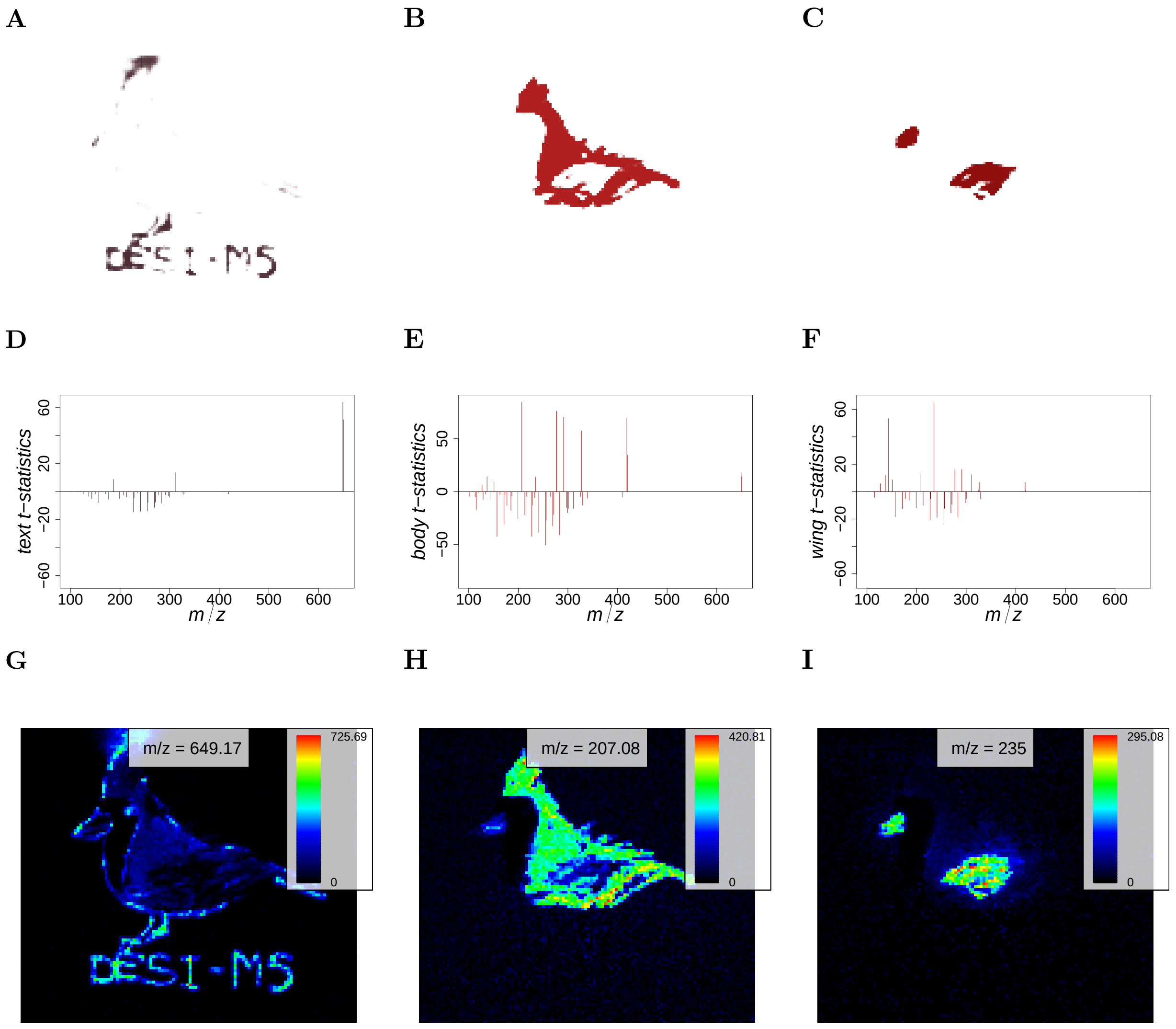

Supplementary Figure 8: **Cardinal painting: t-statistics and representative single ion images.** *A–C* The predicted segment membership probabilites from spatial shrunken centroids with SA distance. *A*, the text segment, *B*, the body segment, and *C*, the wing segment. *D–F* The shrunken t-statistics of the spectral features. *D*, the text segment, *E*, the body segment, and *F*, the wing segment. *G–I* The single ion images corresponding with the top-ranked spectral features by shrunken t-statistic. *G*, the text segment, *H*, the body segment, and *I*, the wing segment.

2.2.4 Classification in supervised experiments

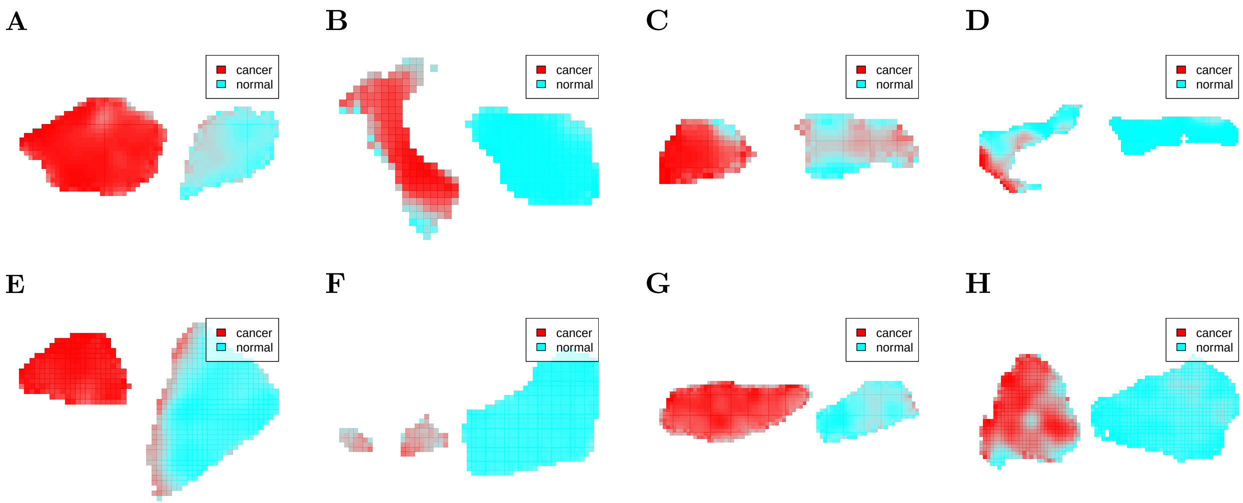

Supplementary Figure 9: **Human renal cell carcinoma: classification.** For each matched pair, cancerous tissue is on the left, and normal tissue is on the right. Transparency is used to show the predicted probabilities based on spatial shrunken centroids classification. The parameters used were $r = 3$, $s = 20$, selected by cross-validation, as illustrated in Supplementary Figure 10.

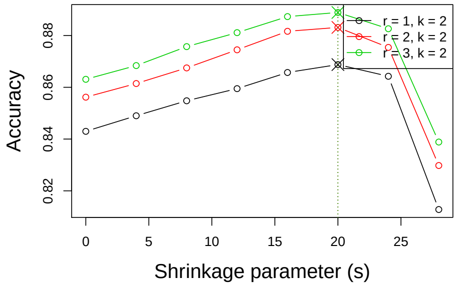

Supplementary Figure 10: **Human renal cell carcinoma: cross-validation.** The highest cross-validated accuracy rate was for $r = 3, s = 20$ with 88.9% accuracy, defined as correctly classifying a pixel as cancer or normal. Each slide was treated as its own fold in 8-fold cross-validation, i.e., leave-one-sample-out cross-validation.

3 References

[1] T. Alexandrov and J. H. Kobarg. Efficient spatial segmentation of large imaging mass spectrometry datasets with spatially aware clustering. *Bioinformatics*, 27:i230, June 2011.

[2] K. D. Bemis, A. Harry, L. S. Eberlin, C. Ferreira, S. M. van de Ven, P. Mallick, M. Stolowitz, and O. Vitek. Cardinal: an R package for statistical analysis of mass spectrometry-based imaging experiments. *Bioinformatics*, 2015.

[3] A. L. Dill, L. S. Eberlin, C. Zheng, A. B. Costa, D. R. Ifa, L. Cheng, T. A. Masterson, M. O. Koch, O. Vitek, and R. G. Cooks. Multivariate statistical differentiation of renal cell carcinomas based on lipidomic analysis by ambient ionization imaging mass spectrometry. *Analytical and Bioanalytical Chemistry*, 398:2969–2978, October 2010.
